# Hypothyroidism increases angiotensinogen gene expression associated with vascular smooth muscle cells cholesterol metabolism dysfunction and aorta remodeling in *Psammomys obesus*

**DOI:** 10.1038/s41598-023-46899-y

**Published:** 2023-11-11

**Authors:** Samia Neggazi, Nadjiba Hamlat, Sihem Berdja, Saliha Boumaza, Leila Smail, Michel Beylot, Souhila Aouichat-Bouguerra

**Affiliations:** 1 Faculty of Biological Sciences, Laboratory of Biology and Physiology of Organisms, Cellular and Molecular Physiopathology team, University of Science and Technology Houari Boumediene, BP 32 El Alia, 16111 Bab Ezzouar, Algiers Algeria; 2 Platform ANIPHY, Faculty of Medicine and Pharmacy, Rockefeller, University Claude Bernard Lyon 1, Lyon, France

**Keywords:** Molecular biology, Biomarkers, Cardiology, Endocrinology

## Abstract

It has been previously shown that clinical cardiovascular manifestations can be caused by mild changes in thyroid function. However, the implication of angiotensinogen (*Agt*) and vascular smooth muscle cells (VSMCs) dysfunction in the pathophysiology of cardiovascular manifestations in hypothyroidism have not yet been investigated. We induced experimental hypothyroidism in *Psammomys obesus* by administering carbimazole for five months. At the end of the experiment, the animals were sacrificed and histopathological analysis was performed using Masson's trichrome staining of the aorta and thyroid gland. The expression of the *Agt* gene and the genes implicated in cholesterol metabolism regulation in the liver and VSMCs was determined by qRT-PCR. Histological observations revealed profound remodeling of the aorta structure in animals with hypothyroidism. In addition, *Agt* gene expression in the liver was significantly increased. In vitro study, showed that VSMCs from hypothyroid animals overexpressed 3-hydroxy-3-methylglutaryl coenzyme A reductase (*Hmgcr*) and Acyl CoA:cholesterol acyltransferase (*Acat*) 1, with failure to increase the efflux pathway genes (ATP-binding cassette subfamily G member (*Abcg) 1 and 4*). These results suggest that hypothyroidism leads to vascular alterations, including structural remodeling, VSMCs cholesterol metabolism dysfunction, and their switch to a synthetic phenotype, together with hepatic *Agt* gene overexpression.

## Introduction

Cardiovascular disease remains the leading cause of death worldwide. It has been previously shown that clinical cardiovascular manifestations can be caused by mild changes in thyroid function^1^. Indeed, subclinical and clinical hypothyroidism have been demonstrated to increase the risk of acceleration and extension of atherosclerosis^2^. In addition, patients with hypothyroidism showed hypercholesterolemia, associated with increased carotid intima-media thickness, and reduced endothelial nitric oxide levels^1^. Moreover, hypothyroidism causes secondary hypertension associated with increased peripheral vascular resistance and arterial stiffness evaluated by the pulse wave velocity (PWV), the gold standard of arterial wall elasticity^3^. Increased PWV is recorded in clinical and even in subclinical hypothyroidism^3^. These clinical features are reversed after adequate hormone replacement therapy^1,3^.

Vascular smooth muscle cells (VSMCs) are the most abundant cells in the vessels and participate in the formation of foam cells in atherosclerotic plaque. It has been demonstrated that more than 50% of foam cells in human and mouse atherosclerotic lesions are SMC-derived following a decrease in the expression of ATP-binding cassette transporter A1 (ABCA1), one of the transporters responsible for cholesterol efflux^4,5^. When exposed to atherogenic lipids and inflammatory factors, SMCs undergo phenotypic modulation associated with reduced expression of specific contractile proteins and increased proliferation and migration abilities^6^. Likewise, thyroid-stimulating hormone (TSH), plays an important part in stimulating the proliferation of VSMCs and the phenotypic change of SMCs from the contractile to the synthetic phenotype^7^. Wang et al.^8^ reported that rat VSMCs dysfunction and apoptosis occur at a very early stage of hypothyroid atherosclerosis, in the absence of demonstrable abnormal endothelial responses. This study further demonstrated that thyroid hormones (TH) protects VSMCs against apoptosis through activating TRα1^8^.

Many animal models have been established and used effectively in cardiovascular disease research. Most of these models are genetically or surgically manipulated^9^. In the current study, we use the sand rat, *Psammomys obesus* a relevant model for cardiovascular pathophysiology^9^ that spontaneously and naturally develop atherosclerotic lesions, when fed on a standard laboratory rodent chow diet^10^. More interestingly, when given a diet rich in cholesterol supplemented with D2 vitamin, *Psammomys obesus* rodents show ulcerated atherosclerotic plaque, resembling human atherosclerotic plaque^11^.

Our study aim to investigate the link between induced hypothyroidism in *Psammomys obesus* model, and the vascular lesions and the role of *Agt* and VSMCs in the vascular remodelling leading to these lesions.

## Results

### Plasma TH levels and thyroid gland histology confirmed hypothyroidism in animals submitted to carbimazole

Animals administered carbimazole showed a significant decrease in plasma concentrations of T_3_, T_4_, FT_3_, and FT_4_ after 3 months (M3) of the experiment, which was maintained at 5 months (M5) (Table 1).

**Table 1:** Thyroid hormones and biochemical parameters at baseline (M0) and after 3 months (M3) and 5 months (M5) of the experiment.

	Control			Hypothyroid		
	M0	M3	M5	M0	M3	M5
T_3_ (nmol/L)	2.1 ± 0.3	2.1 ± 0.3	2.0 ± 0.2	1.8 ± 0.1	1.1 ± 0.04**	0.9 ± 0.04****
T_4_ (nmol/L)	12.8 ± 1.4	11.0 ± 0.7	11.4 ± 1.4	12.3 ± 0.6	6.4 ± 0.9**	4.2 ± 0.7***
FT_3_ (pmol/L)	–	–	–	3.1 ± 0.4	1.5 ± 0.1**	1.2 ± 0.3**
FT_4_ (pmol/L)	–	–	–	13.0 ± 1.7	7.8 ± 0.0.8*	6.6 ± 1.1*
Glucose (mg/dL)	67.0 ± 7.8	59.7 ± 0.9	43.3 ± 3.5	54.8 ± 3.0	46.0 ± 6.0^ns^	36.7 ± 4.4^ns^
Triglycerides (mg/dL)	39.4 ± 4.0	39.0 ± 2.1	30.0 ± 0.6	48.6 ± 6.0	109.7 ± 0.3***	236.7 ± 18.6****
Total cholesterol (mg/dL)	52.0 ± 2.0	60.3 ± 7.3	41.0 ± 7.9	49.8 ± 11.4	78.3 ± 9.8^ns^	96.5 ± 5.4*
Total protein × 10^2^ (mg/dL)	43.4 ± 2.2	44.6 ± 0.7	49.0 ± 2.1	47.2 ± 2.3	47.3 ± 3.7^ns^	55.3 ± 5.0^ns^
% VLDL-LDL-c	40.7 ± 2.2	50.7 ± 0.8	46.7 ± 3.3	44.5 ± 1.3	52.2 ± 0.8^ ns^	58.4 ± 1.3**
% HDL-c	55.5 ± 2.8	50.1 ± 2.2	53.3 ± 3.3	50.1 ± 3.2	35.8 ± 4.1*	33.6 ± 5.0**
% Lp(a)	0	0	0	0	25.0 ± 8.7	30.4 ± 10.6

Values are presented as means ± SEM. Control (n = 6) and hypothyroid group (n = 6) at baseline (M0) and after 3 months (M3) and 5 months (M5) of experiment. FT_3_ means free triidothyronine, FT_4_ free thyroxin, HDL-c high-density lipoprotein cholesterol, LDL-c low-density lipoprotein cholesterol, Lp(a) lipoprotein(a), T_3_ triidothyronine, T_4_ thyroxin, VLDL very low-density lipoprotein levels. Not significant (ns), *p*  ˃ 0.05, *p < 0.05, **p < 0.01, ***p < 0.001, ****p < 0.0001.

**Figure 1:**
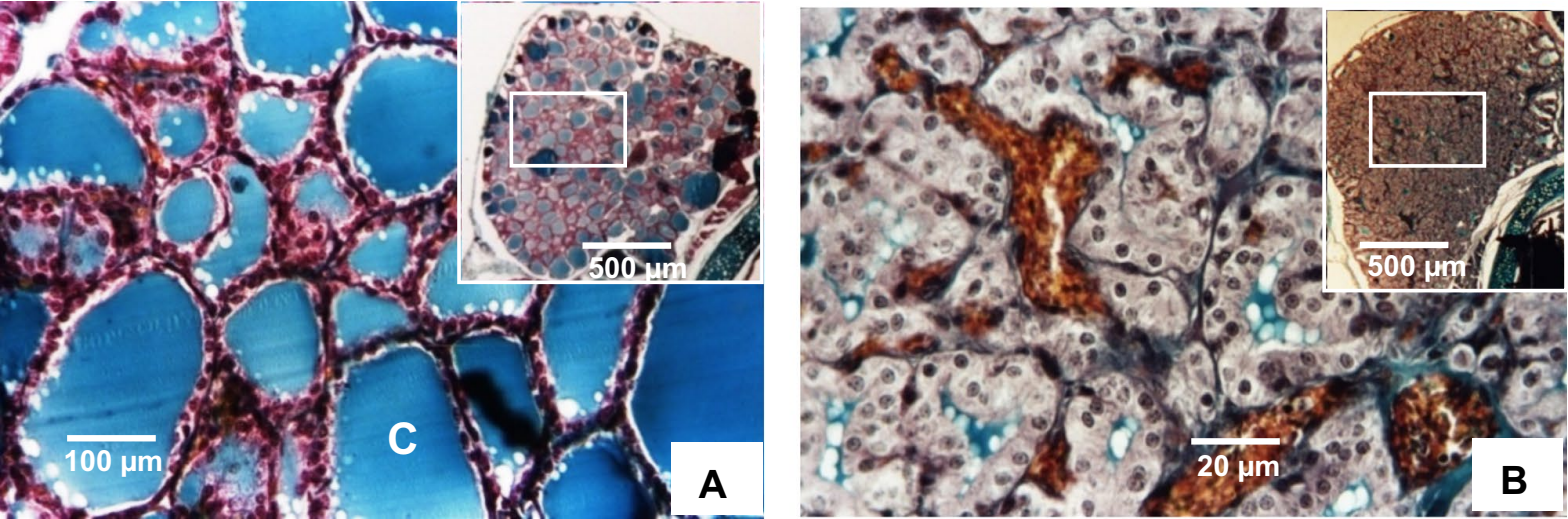
Several follicles of varying sizes and shapes form the thyroid gland of control *Psammomys obesus*, sand rat. A single layer of follicular cells delimited the colloid (C) surrounding the follicle (**A**). Dramatic changes in the morphology of the thyroid gland in *Psammomys obesus* rendered hypothyroid by carbimazole. Follicular hypertrophy and hyperplasia, total absence of colloid, and a significant vascular invasion, with enlargement of these vessels (**B**).

Hypothyroidism was also confirmed by histological examination of the thyroid gland at M5 after the animals were sacrificed. Histological sections of the thyroid glands of the two groups were stained with Masson’s trichrome and examined under a microscope. The thyroid glands of control animals showed numerous follicles of varying sizes and shapes. Each follicle was formed from a single layer of thyrocyte cells, limiting a centrally located colloid (Fig. 1A). In contrast, profound histological alterations were observed in the thyroid glands of animals given carbimazole. Indeed, the thyroid gland showed an increased number of follicles, while their size was decreased. In addition, the cells delimiting the follicles appeared very high, with very reduced and often absent lumina. Interestingly, vascular invasion of the follicles with enlargement of these vessels was also observed (Fig. 1B).

### Hypothyroidism is associated with atherogenic dyslipidemia

Animals submitted to carbimazole for five months showed no changes in plasma total protein concentrations (*p  *˃ 0.05). A trend (but not significant) of lower plasma level of glucose was noted. However, plasma lipid concentrations, including total cholesterol and triglycerides, were significantly increased. The increase in plasma lipid concentrations was accompanied by a significant increase at M5 in the percentage of whole very-low-density lipoprotein (VLDL) and low-density lipoprotein cholesterol (LDL-c) (*p* < 0.01). Simultaneously, the percentage of high-density lipoprotein cholesterol (HDL-c) significantly decreased (*p* < 0.01). Lipoprotein(a) (Lp(a)) was first detected in the hypothyroidism-induced group after M3 and was still found at the end of the experiment (25.0 ± 8.7 and 30.4 ± 10.6%, respectively) (Table 1).

### *Agt* mRNA is overexpressed in the hypothyroid *Psammomys obesus* liver

**Figure 2:**
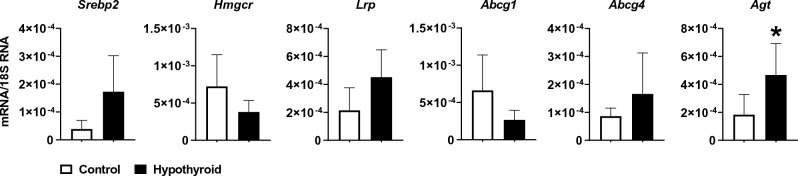
Relative amount of mRNA (ng/µg) normalized to the 18S mRNA level of the genes involved in cholesterol metabolism (*Srebp*2, *Hmgcr*, *Lrp*, *Abcg1*, *Abcg4*) and the *Agt* gene in the liver of the control and hypothyroid *Psammomys obesus* (n = 5 − 6 for each group). The values are presented as means ± SEMs. *p < 0.05 hypothyroid *versus* control group.

qRT-PCR was performed to determine whether hypothyroidism affects the expression of *Agt* mRNA and a set of genes involved in cholesterol metabolism (synthesis, influx, and efflux) in the liver. Interestingly, hypothyroidism in *Psammomys obesus* was associated with a significant increase in *Agt* mRNA levels (*p* < 0.05). In addition, sterol regulatory element-binding protein 2 (*Srebp2*) and LDL-receptor-related protein (*Lrp*) mRNA levels also increased in those animals compared to the control group animals; however, the increase was not statistically significant. In the other hand, 3-hydroxy-3-methylglutaryl coenzyme A reductase (*Hmgcr)* mRNA levels did not significantly decrease. The expression of the genes implicated in cholesterol efflux and influx was not affected (Fig. 2).

### Hypothyroidism induces VSMCs morphology and transcriptomic modifications associated with increased proliferation competence

**Figure 3:**
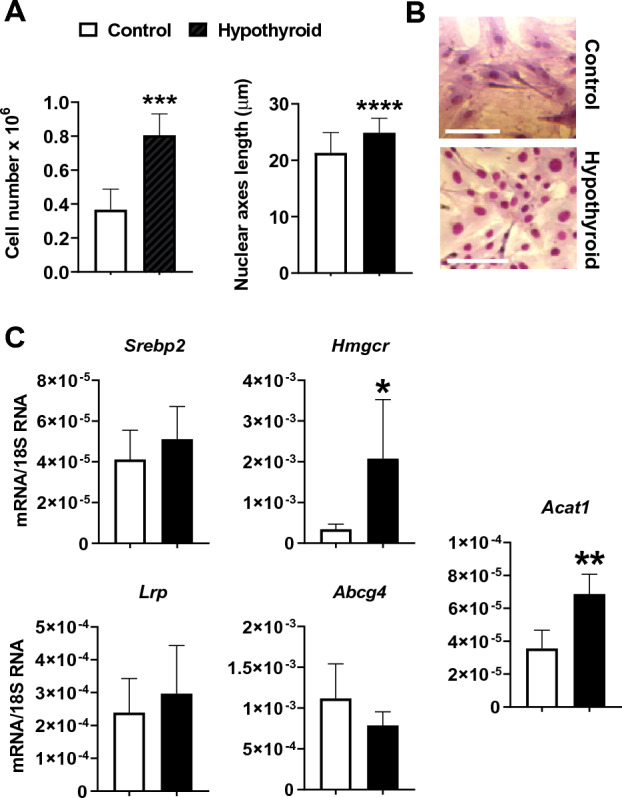
(**A**) Analysis of proliferation (n = 6) and nuclear axis length (n = 100) of control and hypothyroid *Psammomys obesus* VSMCs in secondary culture. (**B**) SMCs of control and hypothyroid *Psammomys obesus* in secondary culture fixed in Bouin’s aqueous and stained with May-Grünwald Giemsa. Scale bar, 50 µm. (**C**) VSMCs mRNA expression (ng/µg) of *Srebp*2, *Hmgcr*, *Lrp*, *Abcg4*, and *Acat1* of control and hypothyroid group (n = 5 − 6 for each group). *Hypothyroid *versus* control VSMCs. *p < 0.05, **p < 0.01, ***p < 0.001, ****p < 0.0001.

A significant increase in the proliferation rate was observed in the VSMCs obtained from the hypothyroid animal group (119.4%) after 72 h of culture compared to the control group (Fig. 3A). The increased rate of proliferation observed in hypothyroid VSMCs prompted us to study the morphometric characteristics of these VSMCs. The cells were then fixed in Bouin’s aqueous solution and stained with May-Grünwald Giemsa stain for morphometric measurements (Fig. 3B). The measurement of the great nuclear axis length showed a highly significant increase in the hypothyroid VSMCs nuclear axes compared to their corresponding controls (24.9 ± 0.3 versus 21.3 ± 0.4 µm) (Fig. 3A).

To determine whether proliferation and morphological changes were associated with changes in transcriptomic activity, we assessed the transcription of five genes involved in VSMCs functions using qRT-PCR. Indeed, at the 4th passage, the hypothyroid VSMCs cultures showed a significant increase in the expression of the *Hmgcr* (*p* < 0.05) and *Acat1* (*p* < 0.01) genes compared to the VSMCs control. However, the expression of the *Srebp*2, *Lrp*, and *Abcg4* was similar in the cultures from the hypothyroid VSMCs and the control group (*p* ˃ 0.05) (Fig. 3C).

### T_3_ stimulates VSMCs *Abcg1* gene expression in the hypothyroid *Psammomys obesus* group

**Figure 4:**
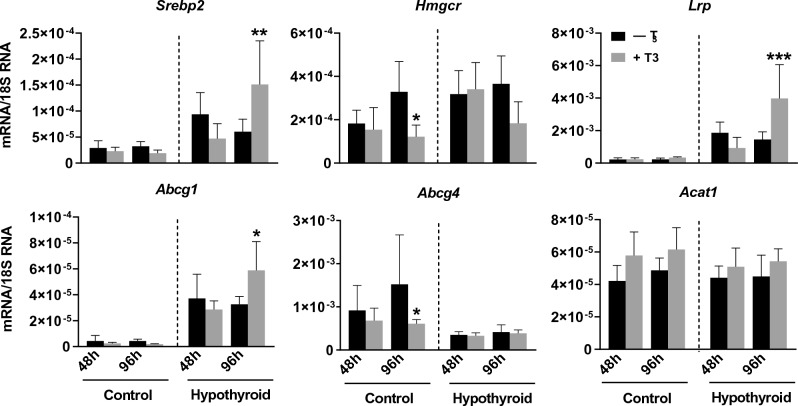
mRNA levels (ng/μg) of genes involved in cholesterol metabolism in VSMCs of the control and hypothyroid group in the absence (– T_3_) and presence of T_3_ (+ T_3_) in the culture medium for 48 h and 96 h. *p < 0.05, **p < 0.01, ***p < 0.001 *versus* the corresponding values in the absence of T_3_.

We subjected the VSMCs derived from the two groups of animals to 1 nM T_3_ added to the culture medium for 48 h and 96 h. After 96 h of incubation with T_3_, VSMCs derived from the control group showed a decrease in *Hmgcr* and ATP-binding cassette subfamily G member 4 (*Abcg4)* expression. In contrast, VSMCs derived from the hypothyroid animal group showed a clear increase in the expression of the gene coding for *Srebp2*, *Lrp*, and *Abcg1* after 96 h of incubation with T_3_ (Fig. 4).

### Cholesterol load significantly increased the cholesterol content in hypothyroid VSMCs

VSMCs have been previously shown to accumulate excess lipids and participate in the total intimal foam cell population, contributing to the development and progression of atherosclerosis^12^. To better understand cholesterol metabolism processes in arterial SMCs, we subjected VSMCs to cholesterol loading in the presence or absence of T_3_. First, cholesterol cell content was measured, and the expression of the genes involved in cholesterol metabolism was assessed.

**Figure 5:**
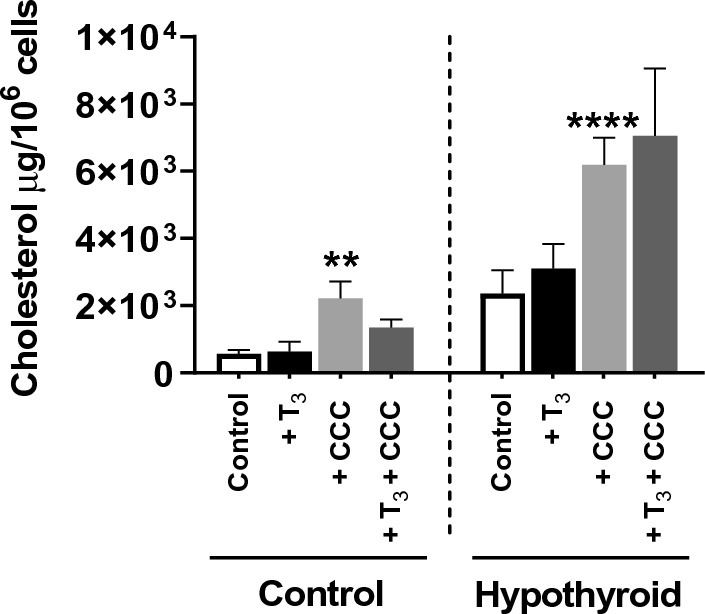
Cholesterol content in VSMCs of the control and hypothyroid *Psammomys obesus*, cultured without (Control) or with T_3_ (+ T_3_), cyclodextrin-cholesterol complexes (+ CCC), and CCC combined with T_3_ (+ T_3_ + CCC). **p < 0.01, ****p < 0.0001 *versus* control.

Before cholesterol loading, we noticed that the cholesterol content in the VSMCs hypothyroid group was significantly higher (fourfold) than that in the VSMCs control group. Incubated in 20 μg/mL of CCC for 48 h, the VSMCs hypothyroid group was likely to load cholesterol even more than the VSMCs control group. Co-loading T_3_ with the cholesterol induced no change in cholesterol content in control and hypothyroid VSMCs (Fig. 5).

**Figure 6:**
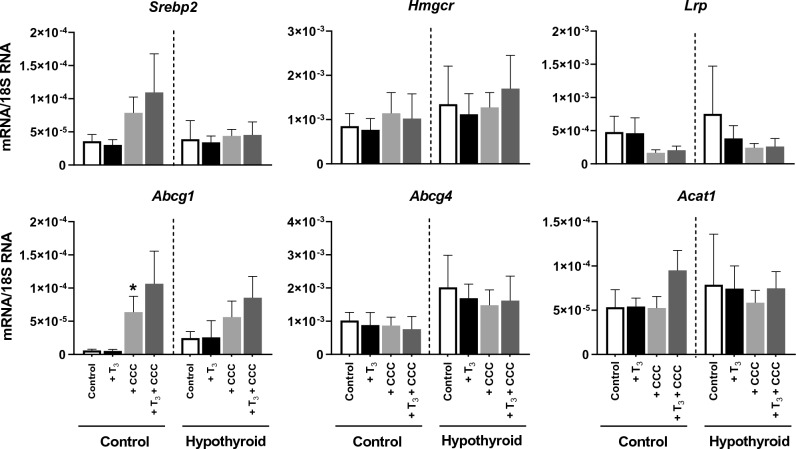
mRNA levels (ng/µg) of *Srebp2*, *Hmgc*r, *Lrp*, *Abcg1*, *Abcg4,* and *Acat1* in VSMCs from control and hypothyroid *Psammomys obesus gerbils* following addition of T_3_ (+ T_3_), CCC (+ CCC), and CCC combined with T_3_ (+ T_3_ + CCC) in the culture medium. The values are presented as means ± SEMs. *versus Control group. *p < 0.05. Hypothyroidism alters the cellular and connective structures of the aortic wall.

The qRT-PCR results showed no significant change in the mRNA levels of genes involved in cholesterol metabolism in VSMCs from the hypothyroid group. However, the cholesterol load induced a significant increase in *Abcg1* mRNA levels in the VSMCs control group (*p* < 0.05) (Fig. 6).

### Hypothyroidism is associated with arterial remodeling

**Figure 7:**
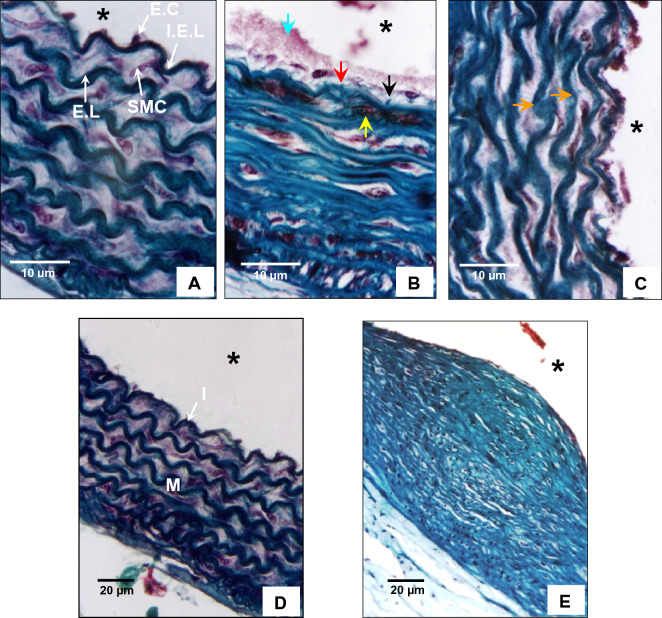
The aortic vascular wall of the control animals was composed of an intima (I) consisting of a single layer of endothelial cells (E.C) with flattened nuclei. The media (M) was formed by smooth muscle cells (SMCs), separated by elastic lamellae (E.L), which appeared very wavy. The media was separated from the intima and adventitia by the internal (I.E.L) and external elastic lamella, respectively (Asterisk black denoted aortic lumen) (**A**,**D**) Endothelial cells are hypertrophied and separated from the I.E.L by a much-thickened subendothelial space containing collagens (black arrow). Thrombus formation (green arrow) and interrupted I.E.L (red arrow) were also observed with infiltration by SMCs in the first interlamellar space, characterized by a synthetic phenotype (yellow arrow) (**B**) Hypothyroidism also caused disorganization of the media, which was manifested by the thinning, ruptures, and fragmentation of E.L (orange arrow) with a decrease in their degree of undulation (**C**). In certain regions of the aortic wall, we observed an increased accumulation of collagens (**E**).

*Psammomys obesus* aortas were stained with Masson’s trichrome to investigate the potential pathological tissue changes in the aortic wall after a decrease in TH. The control group aorta showed an intima composed of the usual endothelial cell layer with flattened nuclei closely apposed to the internal elastic lamella. The media consisted of a set of concentric layers of SMCs separated by elastic lamellae that appeared very wavy in *Psammomys obesus*, delimiting interlamellar spaces (Fig. 7A,D). In contrast, hypothyroidism in *Psammomys obesus* altered the cellular and connective structure of the aortic wall. The endothelial cells were hypertrophied, and were separated from the internal elastic lamella by a much-thickened subendothelial space presenting SMCs and collagen. The internal elastic lamella presented interruption, allowing a direct contact of the circulating blood with cellular and connective elements of the media, and subsequently leads to the formation of thrombus (Fig. 7B). Furthermore, the SMCs no longer show an elongated shape that characterizes their contractile state (Fig. 7B). Finally, the media elastic lamellae lost their undulations and appeared thinner with localized ruptures and fragmentation (Fig. 7C). Increased accumulation of collagen was also observed in the media (Fig. 7E).

## Discussion

Our study contributes to a better understanding of the mechanisms involved in the process of atherogenesis during hypothyroidism. In this study, hypothyroidism was experimentally induced in *Psammomys obesus* by using carbimazole. Thyroid failure, associated with a significant decrease in plasma TH, was successfully induced with 0.03% carbimazole.

Hypothyroidism in *Psammomys obesus* induced significant vascular remodeling, including the thinning of aortic elastic fiber network as well as the loss of lamellae undulation, together with the appearance of localized ruptures and fragmentation. Disorganization of the elastic fiber network was further accompanied by collagen accumulation. More importantly, arterial SMCs have switched from a quiescent contractile to a proliferative synthetic phenotype^13^. These results indicated an increase in the arterial wall stiffness in the hypothyroid group. Arterial wall stiffness was previously shown to be correlated with an increased PWV not only in hypothyroid patients, but also in subclinical hypothyroid patients with normal serum levels of TH and increased serum TSH^14–16^ suggesting the stiffness of the arterial wall to be an early event in hypothyroidism induced-cardiovascular alterations. Furthermore, the stiffness of the arterial wall ultimately leads to an increase in blood pressure, a main risk factor for cardiovascular disease.

Our results further demonstrated that profound remodeling of the aorta wall in *Psammomys obesus* with hypothyroidism was associated with a significant increase in *Agt* gene expression in the liver. This result is in agreement with previous data showing a key role of the renin-angiotensin system in regulating vascular tone and remodeling^17^. Indeed, angiotensin II induces an increase in blood pressure and VSMCs synthetic/secretion activity of extracellular matrix components and proliferation through angiotensin II receptor type 1 (AT1)^17^. These modifications are the main risk factors for atherosclerosis-related cardiovascular diseases. Indeed, it has been demonstrated that inhibition of *Agt* in hepatocytes using genetic and pharmacological manipulations can profoundly reduce systolic blood pressure and atherosclerosis, and *Agt* exerts its effects independently of angiotensin II^18^.

Our current study showed that hypothyroidism induced a slight decrease in glycemia. This decrease may result from impaired glucose absorption in the gastrointestinal tract and its uptake in the peripheral tissues, together with reduced gluconeogenesis and a low glucagon response^19^. Furthermore, hypoglycemia in *Psammomys obesus* with hypothyroidism can be caused by carbimazole intake, which was previously shown to induce insulin autoimmune syndrome in patient with hyperthyroidism^20^.

Hypothyroidism in *Psammomys obesus* caused hypertriglyceridemia, with an increase in total cholesterol and VLDL-LDL-c levels, and a decrease in HDL-c levels. Interestingly, a decrease in TH was also associated with the detection of highly atherogenic Lp(a). Alterations in lipid metabolism, mainly reflected by increased plasma total cholesterol and LDL-c, were commonly described in hypothyroidism^21^. This increase was demonstrated to be the result of increased intestinal absorption of cholesterol in hypothyroidism, and not as a result of increased cholesterol synthesis^22^. The decrease in TH has been shown to inhibit cholesterol synthesis via* Hmgcr* and reduce its uptake via reducing the number of *Ldl*-r in the liver^22^. Increased total plasma cholesterol in hypothyroidism can also be the result of increased hepatic expression of proprotein convertase subtilisin/kexin type 9 (*Pcsk9*) stimulated by serum TSH, which was previously shown to be elevated in subclinical hypothyroidism patients^23^. Our study also showed that hypothyroidism caused a slight increase in *Srebp2* mRNA levels and a slight decrease in *Hmgcr* mRNA levels in the liver. It has been demonstrated that recombinant human TSH (rhTSH) increases expression of *Pcsk9* in HepG2 cells’ cultures dependently of *Srebp2* activation but independently of *Hmgcr*^23^. Our results regarding HDL-c are in contradiction with those obtained by Jung et al.^24^ and by Sigal et al.^25^, who showed that patients who underwent thyroidectomy for thyroid cancer had an increase in HDL-c. However, they showed in the same studies that although HDL-c increased, their atheroprotective function decreased.

Finally, and importantly, our study shows that hypothyroidism affects VSMCs. Indeed, we showed that VSMCs isolated from hypothyroid animals have a significant proliferation rate, as well as an increased length of the great nuclear axes, indicating that these cells switched their phenotype from a quiescent state to a secretory proliferative state. This phenotypic switch was previously described by our team in VSMCs derived from the aorta of diabetes-induced *Psammomys obesus*^26^. Indeed, we found that in the diabetic *Psammomys* model, the phenotype switch was associated with an increase in the total protein secretion, which includes extracellular matrix constituents such as collagens I and III^26^. Our similar observation in the phenotype switch suggests that hypothyroidism can cause similar metabolic alterations, leading to overexpression and secretion of collagens that we observed to deposit in the aorta wall causing its stiffness. In addition, we observed markedly higher cholesterol content in VSMCs derived from hypothyroid animals than in the control-derived VSMCs. This increase could be the result of a disturbance in cholesterol homeostasis following overexpression of the genes promoting its synthesis and esterification (*Hmgc*r and *Acat1*, respectively). In contrast to the observations in control-derived VSMCs, the cholesterol content in hypothyroid-derived VSMCs increased significantly in response to cholesterol loading, with the inability of these cells to increase *Abcg1*-regulated cellular cholesterol efflux. All together, the alterations observed in VSMCs in the context of TH deficiency could contribute additionally to the accelerated development of atherosclerosis and subsequently to an increased risk of cardiovascular disease, the leading cause of death worldwide.

Carbimazole was widely used for hyperthyroidism; however, vascular injury due to this antithyroid therapy was rare. No cases of carbimazole-induced large-vessel vasculitis have been noted. However, rare cases of vasculitis associated with small-vessels have been reported^27,28^. Anti-neutrophil cytoplasmic antibody (ANCA) positivity was rarely associated with carbimazole but a few cases have been reported^29,30^.

In conclusion, our results showed that carbimazole-induced hypothyroidism in *Psammomys obesus* increased *Agt* expression in the liver, with subsequent profound vascular remodeling. Moreover, our in vitro results clearly showed that the deficiency in TH leads to VSMCs quiescence to a synthetic phenotype switch and the dysregulation of cholesterol content in VSMCs, making these VSMCs key players in the development of atherosclerosis.

## Materials and methods

### Biological material and experimental protocol

The experiment was carried out on adult desert rodent of the Gerbillidae family, *Psammomys obesus*. It is a diurnal species that naturally feeding on halophilic plants, rich in water and mineral salts, of the Chenopodiaceae family (especially, *Suaeda mollis*, *Traganum nudatum*, and *Salsola foetida*)^31^. These animals were captured from the Algerian desert. They are very sensitive to climatic changes, the reason why we have tried to achieve appropriate captivity conditions in order to successfully keep it alive^31^. After a period of acclimation at the animal facility, animals were individually housed and divided into two groups as follows: (1) control animals were fed a normal diet of natural halophil plants and water ad libitum; (2) the other group received natural halophil plants plus a synthetic antithyroid drug, carbimazole, dissolved daily in their drinking water at a concentration of 0.03% per animal. At the beginning of the experiment then every month, the animals were bled from the retro-orbital venous plexus; obtained blood samples were immediately centrifuged at 3000 rpm. At the end of the experiment, which lasted five months, the animals were euthanized with urethane (0.8 g/kg) and tissue samples were collected. The thoracic aorta was flushed with isotonic saline and cleaned of any perivascular adipose tissue for VSMCs culture or snap-frozen in liquid nitrogen and kept at − 80 °C for qPCR analysis. Other aortas and thyroid glands were fixed in Bouin’s aqueous solution for histopathological analysis.

### Biochemical analysis

The concentrations of glucose, triglycerides, total cholesterol, and total proteins in plasma samples were measured using BioMérieux kits (France), according to the manufacturer’s instructions. Plasma was used for the lipoproteins assay on agarose gel according to Kawakami et al. method^32^. The plasma triidothyronine (T_3_), free T_3_ (FT_3_), thyroxin (T_4_), and free T_4_ (FT_4_) levels were measured using ELISA kits (Mini Vidas, bioMérieux, France).

### Aortic VSMCs culture

Primary VSMCs were obtained using the explant technique^33,34^. Briefly, explants were prepared after removing the adventitia using collagenase. The small fragments obtained were placed in culture dishes containing DMEM (GIBCO, France) supplemented with penicillin/streptomycin, fungizone, and 10% fetal calf serum and maintained in a humidified CO_2_ incubator at 37 °C until confluence. VSMCs were then trypsinized (Gibco, USA) and subcultured. Cells were seeded at a density of 4 × 10^5^ cells/well in 6-well plates. 24 h before starting any treatment, the culture medium was replaced with basal VSMCs medium with no fetal calf serum. 24 h later, the tested substances were added at the appropriate concentrations in fresh DMEM.

To determine the proliferating rate of VSMCs, primary cultures from the control and hypothyroid groups at the 3rd passage were trypsinized and suspended in DMEM with 10% fetal calf serum, were seeded in 6-well plates at a density of 2 × 10^5^ cells/well. Their proliferation capacities were then estimated 72 h later using Malassez counting cell.

### Effects of TH and cholesterol loading on VSMCs

T_3_ (Sigma, France) was added to the culture medium at a concentration of 1 nM^35^. The cells were incubated with T_3_ for 48 h or 96 h.

To mimic atherosclerotic conditions, VSMCs were loaded with cholesterol using methyl-β-cyclodextrin as a cholesterol-complexing agent, as previously described^33,34^. The cells were incubated for 48 h in DMEM with 0.2% BSA supplemented with 20 μg/mL of cyclodextrin-cholesterol complexes (CCC) alone, or for 48 h in DMEM supplemented with T_3_ alone, and then for 48 h with CCC and T_3_ (1 nM).

### Analysis of the VSMCs’ cholesterol content

Cell lipids were extracted using chloroform–methanol (2:1 v/v)^36^. The chloroform phase was collected, washed with water, and dried under nitrogen. The dried lipids were then dissolved in 100 μL isopropanol. Cholesterol concentration was determined using a cholesterol determination kit, RTU (bioMérieux, France).

### RNA purification and qRT-PCR

Total RNA was isolated from the liver and VSMCs using Trizol® reagent (Invitrogen, France). Reverse transcription of cDNA from the mRNA was performed using Super-Script II TM (Invitrogen). qRT-PCR was performed using iQ SYBR Green Supermix (Bio-Rad), and the reaction was run in a MyIQ thermal cycler (Bio-Rad). To measure the expression of the target genes, a standard range was prepared at the same time as the samples using specific primers for each gene. The amount of mRNA of specific genes was expressed as a ratio *versus* the 18S ribosomal RNA level. The primer sequences are shown in Table 2.

**Table 2:** Primers used for real-time quantitative polymerase chain reaction (qRT-PCR) in the liver and VSMCs from *Psammomys obesus* gerbils.

Gene	Forward primer	Reverse primer
*Srebp2*	cggtcctccatcaacga	gaacgccagacttgtgc
*Hmgcr*	gcccagtggtgcgtctt	ttgcgtcctgccatcgt
*Abcg1*	atgaatcagcgaatgttg	gttctaatgggtgcctct
*Abcg4*	gtatggagcgaggacacc	gcccaggaccaggaagt
*Lrp*	ggtataagcggcgagtc	acatcttgtaggtagggtttc
*Acat1*	ttcctttcgttctttgc	agtttcaccagtccttat
*Agt*	cacctacgttcacttccaagg	gtcactccagtgctggaagttg
18S	tgaggccatgattaagaggg	agtcggcatcgtttatggtc

### Histopathology

For histopathological analysis, the aorta and thyroid gland were fixed in Bouin’s aqueous solution, embedded into paraffin and sectioned at 5 µm thickness. Sections were then stained with Masson’s trichrome^37^.

### Statistical analysis

The PRISM software (GraphPad Prism 8.0, USA) was used for statistical analyses. Normality was verified using the Shapiro–Wilk test. Student’s unpaired *t* test was used to compare two groups, and one-way analysis of variance (ANOVA) with Tukey’s post test was used to compare multiple groups (FT_3_, FT_4_, and Cholesterol load in VSMCs). For Plasma T_3_, T_4_, glucose, triglycerides, total cholesterol, and proteins, as well as the percentage of VLDL-LDL-c and HDL-c concentrations, two-way ANOVA with Sidak’s multiple comparison test was used. All the results were presented as means ± SEMs. Values of *p* < 0.05 were considered statistically significant.

### Ethical approval

All experiments were carried out in accordance to the Institutional Animal Care Committee of the Algerian Higher Education and Scientific Research (DGRSDT; http://www.dgrsdt.dz). The permits and ethical rules had been achieved, and according to the Executive Decree No 10–90 completing the Executive Decree n° 04–82 of the Algerian Government, establishing the terms and approval modalities of animal welfare in animal facilities. The investigation conforms to the ARRIVE guidelines (https://arriveguidelines.org).

## Data Availability

The datasets used and analyzed during the current study are available from the corresponding author on reasonable request.
